# 5α-Androst-3-en-17β-yl acetate

**DOI:** 10.1107/S1600536809053264

**Published:** 2009-12-16

**Authors:** L. C. R. Andrade, J. A. Paixão, M. J. M. de Almeida, E. J. Tavares da Silva, F. M. Fernandes Roleira

**Affiliations:** a CEMDRX, Departamento de Física, Faculdade de Ciências e Tecnologia, Universidade de Coimbra, P-3004-516 Coimbra, Portugal; b Centro de Estudos Farmacêuticos, Laboratório de Química Farmacêutica, Faculdade de Farmácia, Universidade de Coimbra, P-3000-295 Coimbra, Portugal

## Abstract

In the crystal structure of the title compound, C_21_H_32_O_2_, ring *A* is highly distorted, with a conformation inter­mediate between 10β-sofa and 1α,10β-half chair; rings *B* and *C* have slightly flattened chair conformations. Ring *D* assumes an unusual 13β-envelope conformation, probably induced by the acet­oxy substituent. Cohesion of the crystal structure is due only to weak van der Waals inter­actions.

## Related literature

For structure–activity relationships (SAR) of steroids with modified *A* and *D *rings as aromatase inhibitors, see: Cepa *et al.* (2005 ▶, 2008 ▶).  For the synthesis and assignment of the absolute configuration, see: Cepa *et al.* (2008 ▶). For a related structure, see Paixão *et al.* (2001 ▶). For reference bond-length data, see: Allen *et al.* 1987 ▶. For conformational details, see: Duax & Norton (1975 ▶); Cremer & Pople (1975 ▶); Altona *et al.* (1968 ▶).
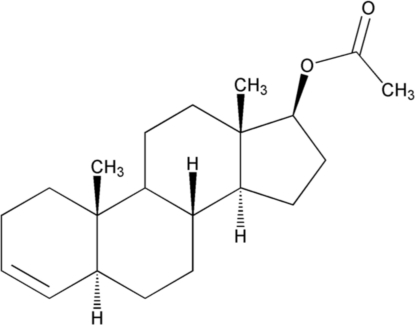


## Experimental

### 

#### Crystal data

C_21_H_32_O_2_
*M* *_r_* = 316.47
Monoclinic, 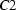

*a* = 14.7728 (3) Å
*b* = 6.2673 (1) Å
*c* = 20.2514 (5) Å
β = 99.874 (1)°
*V* = 1847.21 (7) Å^3^
*Z* = 4
Mo *K*α radiation
μ = 0.07 mm^−1^
*T* = 293 K
0.41 × 0.39 × 0.10 mm

#### Data collection

Bruker APEXII CCD area-detector diffractometer
Absorption correction: multi-scan (*SADABS*; Sheldrick, 2000 ▶) *T*_min_ = 0.971, *T*_max_ = 0.993
22420 measured reflections
2292 independent reflections
1554 reflections with *I* > 2σ(*I*)
*R*_int_ = 0.030

#### Refinement

*R*[*F* ^2^ > 2σ(*F* ^2^)] = 0.045
*wR*(*F* ^2^) = 0.102
*S* = 1.08
2292 reflections
211 parameters
1 restraint
H-atom parameters constrained
Δρ_max_ = 0.13 e Å^−3^
Δρ_min_ = −0.17 e Å^−3^

### 

Data collection: *APEX2* (Bruker, 2003 ▶); cell refinement: *SAINT* (Bruker, 2003 ▶); data reduction: *SAINT*; program(s) used to solve structure: *SHELXS97* (Sheldrick, 2008 ▶); program(s) used to refine structure: *SHELXL97* (Sheldrick, 2008 ▶); molecular graphics: *ORTEPII* (Johnson, 1976 ▶); software used to prepare material for publication: *SHELXL97*.

## Supplementary Material

Crystal structure: contains datablocks I, global. DOI: 10.1107/S1600536809053264/wn2371sup1.cif

Structure factors: contains datablocks I. DOI: 10.1107/S1600536809053264/wn2371Isup2.hkl

Additional supplementary materials:  crystallographic information; 3D view; checkCIF report

## Figures and Tables

**Fig. 1.**
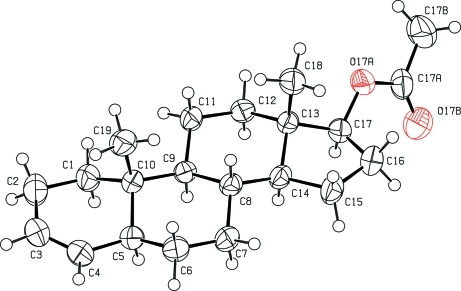
ORTEPII (Johnson, 1976) plot of the title compound. Displacement ellipsoids are drawn at the 50% probability level. Hydrogen atoms are shown as spheres of arbitrary radius.

## supplementary crystallographic information

### Comment

Following our interest in the study of structure-activity relationships (SAR) of steroids with modified A- and D-rings as aromatase inhibitors (Cepa *et al.*, 2005), we have recently prepared and evaluated the title compound (Cepa *et al.*, 2008), the 17-acetyl derivative of a potent aromatase inhibitor previously studied by X-ray diffraction (Paixão *et al.*, 2001). In order to understand the role of specific D-ring substitution patterns in this inhibition process, its crystal structure was determined by single-crystal X-ray diffractometry.

Apart from the C6—C7 bond length, which is slightly shorter [1.511 (3) Å] than the average C*sp*^3^—C*sp*^3^ bond length in the molecule [1.53 (1) Å], bond lengths are well within reported values (Allen *et al.*, 1987). Due to the C3═C4 double bond, ring A is highly distorted with a conformation intermediate between 10β-sofa and 1α,10β-half chair [(asymmetry parameters (Duax & Norton, 1975): ΔC_s_(3)=8.9 (3), ΔC_2_(3,4)=16.8 (4) and ΔC_2_(1,2)=52.2 (4)°]. Rings B and C have slightly flattened chair conformations. Ring D assumes an unusual 13β-envelope conformation [puckering parameters (Cremer & Pople, 1975) q_2_=0.477 (3)Å and φ_2_=188.6 (4)°; pseudo-rotation (Altona *et al.*, 1968) and asymmetry parameters (Duax & Norton, 1975): Δ=25.0 (4), φ_m_=49.3 (2), ΔC_s_(13)=5.8 (3), ΔC_2_(13,14)=17.0 (3) and ΔC_s_(14)=30.5 (3)°). The distance C3—O17B is 11.087 (3) Å. A pseudo-torsion C19—C10···C13—C18 angle of 3.3 (2)° indicates a slight twist of the molecule. The dihedral angle between the least-squares plane of ring D (C14···C17) and that of the four non-H atoms of the 17β acetate group is 69.2 (1)°. The structure lacks any strong hydrogen-bond donor; only weak van der Waals interactions can be responsible for the cohesion of the crystal structure.

### Experimental

The title steroid was prepared according to the procedure previously reported (Cepa *et al.*, 2008), yielding 95% of the pure compound as a white solid. Crystals suitable for X-ray studies were grown by slow evaporation from absolute ethanol. Mp 389–392 K.

### Refinement

All hydrogen atoms were refined as riding on their parent atoms, with C—H = 0.96, 0.97 and 0.98 Å for methyl, methylene and methine hydrogen atoms; *U*_iso_(H) = x*U*_eq_(C), where x = 1.5 for methyl H atoms and 1.2 for all other H atoms. The absolute configuration was known from the synthetic route. In the absence of significant anomalous scattering effects, Friedel pairs were merged.

### Crystal data

**Table:**

C_21_H_32_O_2_	*F*(000) = 696
*M**_r_* = 316.47	*D*_x_ = 1.138 Mg m^−^^3^
Monoclinic, *C*2	Mo *K*α radiation, λ = 0.71073 Å
*a* = 14.7728 (3) Å	Cell parameters from 7585 reflections
*b* = 6.2673 (1) Å	θ = 2.8–21.5°
*c* = 20.2514 (5) Å	µ = 0.07 mm^−^^1^
β = 99.874 (1)°	*T* = 293 K
*V* = 1847.21 (7) Å^3^	Prism, colourless
*Z* = 4	0.41 × 0.39 × 0.10 mm

### Data collection

**Table:**

Bruker APEXII CCD area-detector diffractometer	2292 independent reflections
Radiation source: fine-focus sealed tube	1554 reflections with *I* > 2σ(*I*)
graphite	*R*_int_ = 0.030
φ and ω scans	θ_max_ = 27.9°, θ_min_ = 2.0°
Absorption correction: multi-scan (*SADABS*; Sheldrick, 2000)	*h* = −18→18
*T*_min_ = 0.971, *T*_max_ = 0.993	*k* = −8→8
22420 measured reflections	*l* = −26→25

### Refinement

**Table:**

Refinement on *F*^2^	Primary atom site location: structure-invariant direct methods
Least-squares matrix: full	Secondary atom site location: difference Fourier map
*R*[*F*^2^ > 2σ(*F*^2^)] = 0.045	Hydrogen site location: inferred from neighbouring sites
*wR*(*F*^2^) = 0.102	H-atom parameters constrained
*S* = 1.08	*w* = 1/[σ^2^(*F*_o_^2^) + (0.0455*P*)^2^ + 0.3162*P*] where *P* = (*F*_o_^2^ + 2*F*_c_^2^)/3
2292 reflections	(Δ/σ)_max_ < 0.001
211 parameters	Δρ_max_ = 0.13 e Å^−^^3^
1 restraint	Δρ_min_ = −0.17 e Å^−^^3^

### Special details

**Table:**

Experimental. IR ν_max_(KBr) cm^-1^: 3015 (═C–H), 1733 (C═O); ^1^H NMR (300 MHz, CDCl_3_) δ: 0.77 (3*H*, s, 18-H_3_)*, 0.79 (3*H*, s, 19-H_3_)*, 2.03 (3*H*, s, CH_3_COO), 4.59 (1*H*, dd, *J*_17__α__,16__α_=7.9, *J*_17__α__,16__β_=7.9, 17α-H), 5.27 (1*H*, ddd, *J*_4,3_=9.8, *J*_4,5__α_=4.5, *J*_4,2__α_=2.5, 4-H), 5.54 (1*H*, ddd, *J*_3,4_=9.8, *J*_3,2__β_=6.3, *J*_3,2__α_=3.2, 3-H); ^13^C NMR (75.6 MHz, DMSO-d_6_) δ: 11.8 (C-19)**, 12.2 (C-18)**, 20.5, 21.1, 23.4, 23.5, 27.2, 27.5, 31.5, 34.1, 34.9, 35.3, 36.9, 42.7, 45.8 (C-5), 50.7, 53.3, 82.9 (C-17), 125.4 (C-3), 131.2 (C-4); 171.2 (C═O); EIMS *m/z* 316 (*M*^+^, 100%). *,** Signals may be interchangeable.
Geometry. All e.s.d.'s (except the e.s.d. in the dihedral angle between two l.s. planes) are estimated using the full covariance matrix. The cell e.s.d.'s are taken into account individually in the estimation of e.s.d.'s in distances, angles and torsion angles; correlations between e.s.d.'s in cell parameters are only used when they are defined by crystal symmetry. An approximate (isotropic) treatment of cell e.s.d.'s is used for estimating e.s.d.'s involving l.s. planes.
Refinement. Refinement of *F*^2^ against ALL reflections. The weighted *R*-factor *wR* and goodness of fit *S* are based on *F*^2^, conventional *R*-factors *R* are based on *F*, with *F* set to zero for negative *F*^2^. The threshold expression of *F*^2^ > σ(*F*^2^) is used only for calculating *R*-factors(gt) *etc*. and is not relevant to the choice of reflections for refinement. *R*-factors based on *F*^2^ are statistically about twice as large as those based on *F*, and *R*- factors based on ALL data will be even larger.

### Fractional atomic coordinates and isotropic or equivalent isotropic displacement parameters (Å^2^)

**Table:**

	*x*	*y*	*z*	*U*_iso_*/*U*_eq_	
C1	0.19131 (17)	0.6621 (5)	0.62336 (12)	0.0534 (7)	
H1A	0.1677	0.7480	0.6565	0.064*	
H1B	0.2252	0.7558	0.5983	0.064*	
C2	0.11018 (18)	0.5657 (6)	0.57530 (14)	0.0724 (9)	
H2A	0.0626	0.5259	0.6006	0.087*	
H2B	0.0848	0.6733	0.5429	0.087*	
C3	0.13589 (19)	0.3740 (5)	0.53863 (13)	0.0633 (8)	
H3	0.0923	0.3151	0.5050	0.076*	
C4	0.21769 (18)	0.2854 (5)	0.55206 (12)	0.0525 (7)	
H4	0.2288	0.1648	0.5279	0.063*	
C5	0.29364 (15)	0.3678 (4)	0.60392 (11)	0.0428 (6)	
H5	0.3265	0.4721	0.5808	0.051*	
C6	0.36450 (17)	0.2002 (4)	0.63145 (13)	0.0508 (7)	
H6A	0.3366	0.0950	0.6568	0.061*	
H6B	0.3858	0.1278	0.5946	0.061*	
C7	0.44506 (15)	0.3031 (4)	0.67622 (12)	0.0480 (7)	
H7A	0.4773	0.3944	0.6493	0.058*	
H7B	0.4873	0.1928	0.6958	0.058*	
C8	0.41595 (15)	0.4351 (4)	0.73221 (11)	0.0385 (6)	
H8	0.3911	0.3380	0.7626	0.046*	
C9	0.34047 (14)	0.5989 (4)	0.70474 (10)	0.0353 (5)	
H9	0.3681	0.6951	0.6756	0.042*	
C10	0.25731 (14)	0.4943 (4)	0.65963 (11)	0.0394 (6)	
C11	0.31613 (15)	0.7380 (4)	0.76137 (11)	0.0434 (6)	
H11A	0.2728	0.8468	0.7421	0.052*	
H11B	0.2860	0.6500	0.7905	0.052*	
C12	0.39942 (15)	0.8466 (4)	0.80345 (11)	0.0429 (6)	
H12A	0.4253	0.9487	0.7759	0.052*	
H12B	0.3800	0.9237	0.8401	0.052*	
C13	0.47280 (15)	0.6831 (4)	0.83163 (11)	0.0378 (6)	
C14	0.49663 (14)	0.5547 (4)	0.77246 (11)	0.0399 (6)	
H14	0.5161	0.6592	0.7417	0.048*	
C15	0.58350 (16)	0.4311 (5)	0.80303 (13)	0.0599 (8)	
H15A	0.6212	0.4002	0.7694	0.072*	
H15B	0.5679	0.2981	0.8229	0.072*	
C16	0.63351 (16)	0.5840 (5)	0.85708 (13)	0.0572 (7)	
H16A	0.6450	0.5148	0.9006	0.069*	
H16B	0.6917	0.6301	0.8458	0.069*	
C17	0.56810 (15)	0.7744 (4)	0.85757 (12)	0.0447 (6)	
H17	0.5826	0.8834	0.8263	0.054*	
C18	0.44041 (17)	0.5428 (5)	0.88509 (12)	0.0530 (7)	
H18A	0.4859	0.4358	0.8998	0.080*	
H18B	0.3835	0.4751	0.8664	0.080*	
H18C	0.4316	0.6296	0.9225	0.080*	
C19	0.20421 (17)	0.3490 (5)	0.70079 (13)	0.0562 (7)	
H19A	0.1693	0.4350	0.7265	0.084*	
H19B	0.2468	0.2625	0.7305	0.084*	
H19C	0.1634	0.2587	0.6711	0.084*	
C17A	0.64397 (18)	0.9967 (5)	0.94474 (13)	0.0538 (7)	
C17B	0.6399 (2)	1.0837 (7)	1.01266 (14)	0.0876 (11)	
H17B	0.5804	1.0557	1.0238	0.131*	
H17C	0.6505	1.2348	1.0129	0.131*	
H17D	0.6862	1.0165	1.0450	0.131*	
O17A	0.57234 (11)	0.8688 (3)	0.92319 (8)	0.0531 (5)	
O17B	0.70289 (13)	1.0344 (4)	0.91272 (10)	0.0760 (7)	

### Atomic displacement parameters (Å^2^)

**Table:**

	*U*^11^	*U*^22^	*U*^33^	*U*^12^	*U*^13^	*U*^23^
C1	0.0468 (15)	0.0607 (18)	0.0501 (15)	0.0143 (13)	0.0005 (13)	−0.0053 (14)
C2	0.0503 (16)	0.094 (2)	0.0664 (19)	0.0145 (18)	−0.0084 (15)	−0.021 (2)
C3	0.0517 (17)	0.085 (2)	0.0500 (16)	−0.0041 (17)	−0.0007 (13)	−0.0134 (17)
C4	0.0549 (16)	0.0559 (16)	0.0456 (15)	−0.0013 (14)	0.0061 (13)	−0.0085 (13)
C5	0.0421 (13)	0.0468 (14)	0.0400 (13)	0.0028 (13)	0.0082 (11)	0.0007 (12)
C6	0.0507 (16)	0.0492 (15)	0.0520 (15)	0.0099 (13)	0.0074 (13)	−0.0120 (13)
C7	0.0422 (14)	0.0486 (15)	0.0530 (15)	0.0155 (12)	0.0072 (12)	−0.0062 (13)
C8	0.0365 (12)	0.0400 (13)	0.0394 (13)	0.0057 (11)	0.0079 (10)	0.0030 (11)
C9	0.0356 (12)	0.0359 (13)	0.0354 (12)	0.0034 (11)	0.0085 (10)	0.0034 (11)
C10	0.0324 (12)	0.0456 (14)	0.0399 (13)	0.0033 (11)	0.0059 (10)	0.0007 (11)
C11	0.0379 (13)	0.0472 (16)	0.0437 (14)	0.0117 (12)	0.0028 (11)	−0.0036 (12)
C12	0.0437 (14)	0.0432 (14)	0.0421 (13)	0.0076 (12)	0.0079 (11)	−0.0051 (12)
C13	0.0327 (12)	0.0441 (14)	0.0367 (13)	0.0037 (10)	0.0063 (10)	0.0037 (11)
C14	0.0325 (12)	0.0421 (14)	0.0452 (13)	0.0073 (11)	0.0074 (10)	0.0029 (12)
C15	0.0391 (14)	0.070 (2)	0.0666 (18)	0.0160 (14)	−0.0019 (13)	−0.0073 (16)
C16	0.0378 (13)	0.073 (2)	0.0582 (16)	0.0059 (15)	0.0001 (12)	0.0014 (16)
C17	0.0382 (13)	0.0567 (15)	0.0389 (14)	−0.0024 (12)	0.0059 (11)	0.0019 (12)
C18	0.0490 (15)	0.0644 (17)	0.0459 (15)	−0.0046 (14)	0.0087 (12)	0.0078 (14)
C19	0.0474 (14)	0.0678 (18)	0.0556 (15)	−0.0138 (14)	0.0148 (12)	−0.0053 (15)
C17A	0.0412 (15)	0.0629 (18)	0.0527 (17)	−0.0044 (14)	−0.0049 (13)	0.0013 (14)
C17B	0.070 (2)	0.124 (3)	0.0644 (19)	−0.028 (2)	0.0012 (15)	−0.027 (2)
O17A	0.0442 (10)	0.0721 (12)	0.0418 (9)	−0.0114 (10)	0.0045 (7)	−0.0088 (10)
O17B	0.0516 (11)	0.1011 (17)	0.0758 (14)	−0.0238 (13)	0.0125 (10)	−0.0063 (13)

### Geometric parameters (Å, °)

**Table:**

C1—C2	1.533 (3)	C11—H11B	0.9700
C1—C10	1.533 (3)	C12—C13	1.529 (3)
C1—H1A	0.9700	C12—H12A	0.9700
C1—H1B	0.9700	C12—H12B	0.9700
C2—C3	1.495 (4)	C13—C17	1.527 (3)
C2—H2A	0.9700	C13—C18	1.534 (3)
C2—H2B	0.9700	C13—C14	1.534 (3)
C3—C4	1.315 (3)	C14—C15	1.536 (3)
C3—H3	0.9300	C14—H14	0.9800
C4—C5	1.491 (3)	C15—C16	1.545 (4)
C4—H4	0.9300	C15—H15A	0.9700
C5—C6	1.520 (3)	C15—H15B	0.9700
C5—C10	1.548 (3)	C16—C17	1.536 (4)
C5—H5	0.9800	C16—H16A	0.9700
C6—C7	1.511 (3)	C16—H16B	0.9700
C6—H6A	0.9700	C17—O17A	1.446 (3)
C6—H6B	0.9700	C17—H17	0.9800
C7—C8	1.524 (3)	C18—H18A	0.9600
C7—H7A	0.9700	C18—H18B	0.9600
C7—H7B	0.9700	C18—H18C	0.9600
C8—C14	1.521 (3)	C19—H19A	0.9600
C8—C9	1.547 (3)	C19—H19B	0.9600
C8—H8	0.9800	C19—H19C	0.9600
C9—C11	1.532 (3)	C17A—O17B	1.195 (3)
C9—C10	1.546 (3)	C17A—O17A	1.340 (3)
C9—H9	0.9800	C17A—C17B	1.490 (4)
C10—C19	1.538 (3)	C17B—H17B	0.9600
C11—C12	1.531 (3)	C17B—H17C	0.9600
C11—H11A	0.9700	C17B—H17D	0.9600
			
C2—C1—C10	113.4 (2)	H11A—C11—H11B	107.7
C2—C1—H1A	108.9	C13—C12—C11	111.2 (2)
C10—C1—H1A	108.9	C13—C12—H12A	109.4
C2—C1—H1B	108.9	C11—C12—H12A	109.4
C10—C1—H1B	108.9	C13—C12—H12B	109.4
H1A—C1—H1B	107.7	C11—C12—H12B	109.4
C3—C2—C1	113.2 (2)	H12A—C12—H12B	108.0
C3—C2—H2A	108.9	C17—C13—C12	115.5 (2)
C1—C2—H2A	108.9	C17—C13—C18	110.66 (18)
C3—C2—H2B	108.9	C12—C13—C18	111.13 (19)
C1—C2—H2B	108.9	C17—C13—C14	98.27 (17)
H2A—C2—H2B	107.7	C12—C13—C14	107.64 (17)
C4—C3—C2	122.6 (3)	C18—C13—C14	113.1 (2)
C4—C3—H3	118.7	C8—C14—C13	114.43 (17)
C2—C3—H3	118.7	C8—C14—C15	119.4 (2)
C3—C4—C5	123.6 (3)	C13—C14—C15	103.75 (18)
C3—C4—H4	118.2	C8—C14—H14	106.1
C5—C4—H4	118.2	C13—C14—H14	106.1
C4—C5—C6	114.0 (2)	C15—C14—H14	106.1
C4—C5—C10	112.18 (18)	C14—C15—C16	103.8 (2)
C6—C5—C10	112.93 (18)	C14—C15—H15A	111.0
C4—C5—H5	105.6	C16—C15—H15A	111.0
C6—C5—H5	105.6	C14—C15—H15B	111.0
C10—C5—H5	105.6	C16—C15—H15B	111.0
C7—C6—C5	110.3 (2)	H15A—C15—H15B	109.0
C7—C6—H6A	109.6	C17—C16—C15	105.17 (18)
C5—C6—H6A	109.6	C17—C16—H16A	110.7
C7—C6—H6B	109.6	C15—C16—H16A	110.7
C5—C6—H6B	109.6	C17—C16—H16B	110.7
H6A—C6—H6B	108.1	C15—C16—H16B	110.7
C6—C7—C8	112.61 (19)	H16A—C16—H16B	108.8
C6—C7—H7A	109.1	O17A—C17—C13	111.19 (17)
C8—C7—H7A	109.1	O17A—C17—C16	113.30 (19)
C6—C7—H7B	109.1	C13—C17—C16	104.5 (2)
C8—C7—H7B	109.1	O17A—C17—H17	109.2
H7A—C7—H7B	107.8	C13—C17—H17	109.2
C14—C8—C7	111.78 (18)	C16—C17—H17	109.2
C14—C8—C9	108.42 (18)	C13—C18—H18A	109.5
C7—C8—C9	111.75 (17)	C13—C18—H18B	109.5
C14—C8—H8	108.3	H18A—C18—H18B	109.5
C7—C8—H8	108.3	C13—C18—H18C	109.5
C9—C8—H8	108.3	H18A—C18—H18C	109.5
C11—C9—C10	114.78 (17)	H18B—C18—H18C	109.5
C11—C9—C8	110.83 (16)	C10—C19—H19A	109.5
C10—C9—C8	112.44 (17)	C10—C19—H19B	109.5
C11—C9—H9	106.0	H19A—C19—H19B	109.5
C10—C9—H9	106.0	C10—C19—H19C	109.5
C8—C9—H9	106.0	H19A—C19—H19C	109.5
C1—C10—C19	108.93 (19)	H19B—C19—H19C	109.5
C1—C10—C9	111.59 (19)	O17B—C17A—O17A	123.3 (3)
C19—C10—C9	111.01 (18)	O17B—C17A—C17B	125.3 (3)
C1—C10—C5	105.87 (18)	O17A—C17A—C17B	111.4 (2)
C19—C10—C5	111.4 (2)	C17A—C17B—H17B	109.5
C9—C10—C5	107.95 (16)	C17A—C17B—H17C	109.5
C12—C11—C9	113.60 (18)	H17B—C17B—H17C	109.5
C12—C11—H11A	108.8	C17A—C17B—H17D	109.5
C9—C11—H11A	108.8	H17B—C17B—H17D	109.5
C12—C11—H11B	108.8	H17C—C17B—H17D	109.5
C9—C11—H11B	108.8	C17A—O17A—C17	116.84 (19)
			
C10—C1—C2—C3	−37.9 (3)	C9—C11—C12—C13	−55.1 (3)
C1—C2—C3—C4	6.9 (4)	C11—C12—C13—C17	163.78 (18)
C2—C3—C4—C5	−1.2 (5)	C11—C12—C13—C18	−69.1 (2)
C3—C4—C5—C6	155.6 (3)	C11—C12—C13—C14	55.2 (2)
C3—C4—C5—C10	25.7 (4)	C7—C8—C14—C13	−177.5 (2)
C4—C5—C6—C7	172.2 (2)	C9—C8—C14—C13	58.9 (2)
C10—C5—C6—C7	−58.2 (3)	C7—C8—C14—C15	−53.7 (3)
C5—C6—C7—C8	54.7 (3)	C9—C8—C14—C15	−177.28 (19)
C6—C7—C8—C14	−174.4 (2)	C17—C13—C14—C8	179.97 (19)
C6—C7—C8—C9	−52.7 (3)	C12—C13—C14—C8	−59.9 (2)
C14—C8—C9—C11	−53.2 (2)	C18—C13—C14—C8	63.3 (2)
C7—C8—C9—C11	−176.8 (2)	C17—C13—C14—C15	48.1 (2)
C14—C8—C9—C10	176.82 (17)	C12—C13—C14—C15	168.3 (2)
C7—C8—C9—C10	53.2 (2)	C18—C13—C14—C15	−68.6 (2)
C2—C1—C10—C19	−60.0 (3)	C8—C14—C15—C16	−162.0 (2)
C2—C1—C10—C9	177.1 (2)	C13—C14—C15—C16	−33.2 (2)
C2—C1—C10—C5	59.9 (3)	C14—C15—C16—C17	4.6 (3)
C11—C9—C10—C1	61.9 (2)	C12—C13—C17—O17A	78.3 (3)
C8—C9—C10—C1	−170.18 (18)	C18—C13—C17—O17A	−49.0 (3)
C11—C9—C10—C19	−59.8 (3)	C14—C13—C17—O17A	−167.57 (19)
C8—C9—C10—C19	68.1 (2)	C12—C13—C17—C16	−159.1 (2)
C11—C9—C10—C5	177.85 (19)	C18—C13—C17—C16	73.6 (2)
C8—C9—C10—C5	−54.2 (2)	C14—C13—C17—C16	−45.0 (2)
C4—C5—C10—C1	−52.5 (3)	C15—C16—C17—O17A	146.8 (2)
C6—C5—C10—C1	177.1 (2)	C15—C16—C17—C13	25.6 (3)
C4—C5—C10—C19	65.8 (3)	O17B—C17A—O17A—C17	−0.1 (4)
C6—C5—C10—C19	−64.7 (2)	C17B—C17A—O17A—C17	179.8 (2)
C4—C5—C10—C9	−172.1 (2)	C13—C17—O17A—C17A	−164.9 (2)
C6—C5—C10—C9	57.5 (2)	C16—C17—O17A—C17A	77.7 (3)
C10—C9—C11—C12	−177.70 (18)	C19—C10—C13—C18	3.33 (19)
C8—C9—C11—C12	53.6 (2)		
